# Orthopaedic surgeons’ perceptions of frailty and frailty screening

**DOI:** 10.1186/s12877-019-1404-8

**Published:** 2020-01-16

**Authors:** Mandy M. Archibald, Michael Lawless, Tiffany K. Gill, Mellick J. Chehade

**Affiliations:** 1 for the Centre of Research Excellence in Frailty and Healthy Ageing, Adelaide, Australia; 2 University of Manitoba, College of Nursing, 99 Curry Pl, Winnipeg, MB R3T 2M6 Canada; 3 Flinders University, College of Nursing and Health Sciences, Bedford Park, SA 5042 Australia; 4 Adelaide Medical School, South Australian Health and Medical Research Institute, Level 7, North Tce, Adelaide, SA 5000 Australia; 5 Discipline of Orthopaedics & Trauma, Royal Adelaide Hospital, The University of Adelaide, Level 4 Bice Building, Adelaide, SA 5005 Australia

**Keywords:** Frailty, Frailty screening, Qualitative, Orthopedics, Fragility

## Abstract

**Background:**

Over the past decade, there has been significant growth in the awareness and understanding of fragility among orthopaedic surgeons in the context of osteoporotic fractures and with it, improvements in the recognition and management of fragility fractures. Emerging as a major clinical and research focus in aged care is the concept of frailty and its associations with fragility, sarcopenia, falls and rehabilitation. Currently, research is lacking on how orthopaedic surgeons perceive frailty and the role of frailty screening. A baseline understanding of these perceptions is needed to inform integration of frailty identification and management for patient optimization in orthopaedic practices, as well as research and education efforts of patients and healthcare professionals in orthopaedic contexts.

**Methods:**

We used an exploratory design guided by qualitative description to conduct 15 semi-structured telephone and in-person interviews across three orthopaedic surgeon subgroups (Registrars, Junior Consultants, and Senior Consultants). Data collection and analysis occurred iteratively and was guided by thematic saturation.

**Results:**

Orthopaedic surgeons have a disparate understanding of frailty. Between colleagues, frailty is often referred to non-specifically to suggest a general state of risk to the patient. Frailty screening is regarded positively but its specific utility in orthopaedic environments is questioned. Easy-to-administer frailty screening tools that are not exclusive assessments of functional status are viewed most satisfactorily. However these tools are rarely used.

**Conclusions:**

There is little understanding among orthopaedic surgeons of frailty as a phenotype. Beliefs around modifiability of frailty were dissimilar as were the impact of related risk factors, such a cognitive status, chronic disease, social isolation, and environmental influences. This in turn may significantly impact on the occurrence and treatment outcomes of fragility fracture, a common orthopaedic problem in older populations. This study highlights need for knowledge translation efforts (e.g. education) to achieve cohesive understanding of frailty among health professionals.

## Background

Growing numbers of frail older adults are being admitted to hospital with complex health problems and acute care needs. While definitions vary, frailty is commonly described in the medical literature as a clinically recognisable state of increased susceptibility to adverse health outcomes following a stressor event, predisposing individuals to disability, hospitalisation, institutionalisation, and premature death [1–3]. While frailty is a multidimensional concept as reflected in the frailty index approach to measurement, it is often operationalized using clinical indicators, or phenotypic markers, such as reduced muscle strength, unintended weight loss, low physical activity, fatigue, and impairment of physical function [4, 5]. Moreover, frail older patients typically have lower bone mineral density [6] and lower lean body mass [7] than non-frail patients. These mechanisms and others can cause weakness, unsteady gait, and impaired balance, thereby increasing patients’ susceptibility to falls, fragility fractures, and subsequent mortality [8].

Estimates of the prevalence of frailty range from 4.9 to 27.3%, depending on the region and the measurement instrument used, with the prevalence of pre-frailty– a “clinically silent” intermediate stage between non-frail/robust and frail– ranging from 34.6 and 50.9% [9]. Like many countries, Australia’s population of older adults is expanding rapidly. It has been estimated that by 2031, 3.9 million Australians aged 65 years and over will be either frail or at-risk of becoming frail [10]. As a result, support for the clinical operationalization of frailty is growing, including policy and practice recommendations to screen older adults for frailty using validated measurement tools. However, the widespread adoption of these tools across practice settings has not yet been achieved. A critical component to optimizing frailty identification and management in clinical practice is generating a baseline understanding of how acute care providers perceive frailty and frailty screening, considerations that to date have received insufficient attention in the literature [11–13].

Orthopaedic surgeons routinely treat older patients presenting with fragility fractures (e.g., hip, spine, wrist). These patients typically experience complex health problems, comorbidity, and/or higher degrees of dependence in activities of daily living [14]. Individuals with higher levels of frailty in such acute care settings are challenged to overcome the additional physiological challenges posed by trauma and subsequent surgery [15]. For instance, frailty status predicts increased intra-operative resource use and post-operative care requirements following revision hip surgery, including higher vasopressor support needs and length of stay [16], and a reduced chance of returning home within 30 days after hip fracture [17]. Systematic and objective identification of frailty has the potential to improve clinical decision making related to post operative functional recovery, helping predict the benefits and risks of surgical intervention [17]. Frail older persons are vulnerable to sudden and dramatic changes in health and medical complications (e.g., delirium, urinary tract infection, sepsis) triggered by seemingly insignificant events such as a change in medication, and infection [18, 19], or more notable events, such as surgical procedures. The ability of acute care providers to adequately prepare for, recognize and respond to the needs of frail older adults is paramount to aiding prognosis and care plan optimization [20].

In a 2012 position statement, the American College of Surgeons and the American Geriatrics Society recommended that frailty screening be conducted as a component of the pre-operative assessment of older surgical candidates [21].

Yet, providers’ awareness of frailty and perception of its importance to intra and post operative (and ideally, pre-operative) surgical care, as well as the availability and suitability of frailty screening tools for orthopaedic practice settings, are critical and often-overlooked pre-cursors to the adoption of such best practices in the care of older adults with frailty [13]. While a number of qualitative studies have examined perceptions of frailty and frailty screening among the public [13, 18], health care provider groups including general practitioners [22], and healthcare policy-makers [23], we are unaware of any study exploring orthopaedic surgeons’ perceptions and attitudes towards frailty and frailty screening. Given the growing numbers of frail older people attending hospital with complex care needs, the increased surgical demands in this population [16], and the potential clinical impact of frailty screening in outcome optimization for orthopaedic patients, research is needed to understand provider perspectives of frailty and frailty screening in order to identify barriers to optimal frailty management. The current study responds to this gap by exploring orthopaedic surgeons perceptions of frailty and frailty screening in the South Australian practice context.

## Methods

### Aim, Design & Sample

We aimed to understand orthopaedic surgeons’ perceptions and attitudes towards frailty and frailty screening. Using an exploratory qualitative descriptive design [24], we compared the perspectives of three orthopaedic subgroups of Registrars, Junior (Jr.) Consultants, and Senior (Sr.) Consultants to elucidate opportunities and barriers associated with identifying and managing frail older patients in orthopaedic practice to then help inform knowledge translation efforts (e.g., staff education, new resource allocation models) aimed at achieving a systematic approach to recognising and responding to frailty in acute care and across care transitions. This study is part of a larger body of research investigating the perceptions of health care providers and the public towards frailty and frailty screening, in order to help inform knowledge translation strategies for robust, pre-frail, and frail older persons in South Australia [13], and is linked to other Centre of Research Excellence studies exploring the feasibility and diagnostic test accuracy of commonly used frailty screening instruments [25]. Additional details of the study context, affiliated qualitative study arms, and research methods are available in our research protocol [13].

We used a criterion-based approach to purposively sample orthopaedic Registrars, Jr. Consultants, and Sr. Consultants from a large metropolitan city in South Australia (population 1.3 million). We defined Registrars as those who had not yet completed their examination requirements at the time of the interview, Jr. Consultants as those who had completed their examinations requirements within the past 10 years, and Sr. Consultants as those who have been practicing as a consultant for over 10 years. Participants were identified using MJC’s established clinical and medical education network and contacted over email with study information and a request to schedule an interview. Follow up emails were provided to non-responders.

### Data collection and analysis

Data were collected between March and April 2017. Participants provided verbal or written consent to participate, answered brief demographic questions, and participated in a semi-structured telephone or in-person interview (Additional file 1); data collection methods were chosen depending on participant preference and availability. A researcher experienced in qualitative methodology conducted all interviews. Participants and the interviewer were not known to each other prior to the study. Sampling and analysis occurred iteratively and informed sample size.

Semi-structured in person and telephone interviews were conducted. The interview guide was developed based on current understanding of the literature and the research objectives of a Centre for Research Excellence in Frailty and Healthy Ageing (https://health.adelaide.edu.au/cre-frailty/) as detailed in our study protocol [13]. The first half of the interview focused on perceptions of frailty (e.g., the meaning of frailty; the clinical usefulness of frailty; perspectives on prevention and reversibility, for example). The second half of the interview focused on perceptions of frailty screening and a feasibility review of seven validated frailty screening tools (i.e., Edmonton Frail Scale, Groningen Frailty Index (GFI), PRISMA-7, Gait Speed, Timed Up and Go, The Frail Questionnaire, The Kihon Checklist). These specific tools were selected as part of larger program of work including a feasibility and diagnostic test accuracy study of commonly used frailty screening instruments [25], and subsequent qualitative studies with healthcare provider and consumer groups [13, 22]. As part of the selection process, a literature review was conducted; 14 tools were shortlisted for deliberation in reference to the tools’ validity (i.e., minimum sensitivity of 0.6); contextual appropriateness (i.e., English, relevant to Australian practice context); time to implement (i.e., < 20 min), and administration method (i.e., administered rather than based on health records) [25]. Participants were provided the tools over email in advance of the interview. During the interview, each participant was provided with a brief summary of the general features of each tool. They were asked to comment on the tools perceived advantages and disadvantages and then rank them in order of preference considering the unique features of their practice context. This general and non-directive approach to ranking allowed for each participant to identify and prioritize based on factors relevant to each practice context (e.g., time to administer, suitability to the specific clinical population served). Interviews progressed from the general (e.g., what does frailty mean to you) to more structured and specific questions. Reflective notes were taken with each interview to guide questioning and aid analysis.

Interviews were audio recorded, professionally transcribed verbatim, and managed using Microsoft Excel. Inductive analysis occurred iteratively with data collection and generally followed four steps. First, transcripts were read repeatedly and data cleaned to gain a broad sense of the data. Second, MA devised a preliminary coding structure consisting of 63 categories, based on her familiarity with the data. Analysts then extracted and coded data into a Microsoft excel workbook and iteratively revised the coding framework as new codes were identified. When new codes were identified, previous narrative data was revisited to ensure comprehensiveness and accuracy with coding in a manner consistent with the constant comparative approach. Codes were then loosely grouped into categories with the entire sample as unit of analysis; qualitatively extracted data was open coded and numerical codes were tabulated to help identify patterns in the data, including within and between group similarities and differences (Registrar, Jr. and Sr. Consultants). Third, categories were described narratively in a descriptive summary and two analysts (MA, ML) used this to independently identify themes. Tentative themes were discussed and agreed upon through deliberation and in reference to supportive data. Analytic rigor was promoted through analysis meetings where codes, categories, tabulations, and themes were cross-examined, and by the use of analytic memos for audit and reflection. Particular credence was given to the clinical relevance of findings within orthopaedic practice contexts.

When participants were asked to rank the frailty screening tools according to their preference, we tallied these ranks in order. We also calculated a composite score for each screening tool. This involved inverting the numerical ranking ascribed by participants to each tool and tabulating these, resulting in the lowest composite number representing the most desirable tool.

## Results

Fifteen orthopaedic Registrars or Pre-Registrars (*n* = 6), Jr. Consultants (*n* = 4), and Sr. Consultants (*n* = 5) participated in semi-structured interviews (*n* = 12 telephone and *n =* 3 in-person). Interview length ranged from 20.5 min – 57.3 min (*M* = 34.0 min). Fourteen of the participants (93%) were male. A majority of the participants (87%) worked within the public sector at major tertiary hospitals in the Adelaide metropolitan region. These participants held the position of Pre-Registrar or Registrar, Medical Fellow, Specialist, Head of Unit /Department, or Medical Officer. One participant worked in both the private and public sectors; one participant was an upper limb specialist working in private practice. The age range of participants in the sample was between 29 and 72 years. Years in practice ranged from less than 1 (i.e., one Registrar entering first year of orthopaedics training program) to 40.

We inductively developed four main themes. First, participants described frailty as a complex and age related multidimensional state involving physical and mental components. They described the development of frailty as a “vicious cycle” with a number of contributing factors and conditions. Second, frailty was regarded as a familiar term but with a context dependent meaning. Participants described associations of frailty with fragility, and identified differences in general (e.g., colloquial) and medical understandings and uses of the term. Third, frailty was understood as preventable and reversible, but only under certain conditions. Participants identified relevant single (e.g., exercise) and bundled (e.g., exercise and nutrition support) programs pertinent to frailty management. Fourth, participants recognised that formal screening has utility, but its value in orthopaedic practices was unclear. Participants described alternative strategies to identifying frail older persons, possible benefits of screening, and desirable characteristics of screening tools for frailty relevant to orthopaedic practice environments.

### Theme 1: frailty was described as a complex and age related multidimensional state of risk involving physical and mental components

Orthopaedic surgeons in our sample generally understood frailty as a complex, age-related, and multi-dimensional condition involving physical and mental components associated with increased dependency. The majority regarded frailty as a state of risk (80%) involving physical and mental components (12/15, 80%), and 80% regarded frailty as a complex, multidimensional condition. Registrars were less likely than Jr. and Sr. consultants to identify the multidimensional nature and mental and physical components of frailty. Although all Jr. and Sr. consultants recognized frailty as involving multiple body systems (e.g, Jr. Consultant, P7; Sr. Consultants P1, 3–6), and incorporating both social and physiologic factors (e.g., Jr. Consultants, P8, 13), registrars were more likely to exclusively emphasize physiologic components, such as muscle weakness and loss of bone density (P9), in their descriptions of frailty. Such a sentiment was exemplified by a registrars’ response to defining frailty as, “generally, the elderly population is physically and physiologically weaker compared to the normal population” (P14).

“Risk” was commonly associated with frailty and was discussed generally. Frail persons were seen as being at risk for numerous conditions and negative outcomes, such as injury (Sr. Consultant, P5), or “a multitude of medical problems” (e.g., Jr. Consultant, P7). Approximately 33% of participants explicitly identified that frailty increases risk of falls. However, risk was more often discussed generally, as expressed by a Jr. Consultant: “it is not exactly a positive state either so it means you are at risk of having problems in every sense probably” (P2). All Registrars (6/6, 100%) and the majority of Jr. (3/4, 75%) and Sr. (4/5, 80%) understood frailty as associated with risk, with another Jr. Registrar (P13) identifying an increased risk for adverse outcomes as a result of factors influencing frailty, such as vitamin D deficiency.

Participants identified numerous contributing factors associated with the development of frailty. Age was the predominant contributing factor identified by 14 of 15 participants (93%), followed by inactivity (8/15, 53%) and nutrition (7/15, 47%). Approximately 25% of participants described mental status and social support as important contributing factors. Social and environmental factors were identified as central to the occurrence and progression of frailty. Only two Sr. Consultants identified the role of individual attitude in the progression of frailty; a decision to become frail was described by one Sr. Consultant (P3) who drew extensively on his clinical practice to illustrate the relationship between the frailty identity and individual behavior.

Frailty was regarded as related to, but distinct from, common conditions and geriatric syndromes pertinent to ortho-geriatric practice. For instance, sarcopenia, osteoporosis, and fragility are commonly encountered but orthopaedic surgeons acknowledged the breadth and multisystem impact of frailty. As one Sr. Consultant expressed, “As orthopaedic surgeons, we’ve always discussed osteoporosis and sarcopenia and all sorts of things by themselves, but I think frailty is more a complex of an elderly patient that has multiple issues that are part of getting older” (P1).

Frailty as a “vicious cycle” was the most common model of frailty presented and was described by 33% of participants, including 33% of Registrars, 50% of Jr. Consultants, and 17% of Sr. Consultants. No participants described the onset of frailty as a sudden occurrence. A further 27% of participants described frailty as a natural part of ageing, and 13% of participants (*n* = 2) – both Registrars – viewed frailty as an inevitable aspect of ageing. These same two Registrars (P9 & P11) characterised frailty both as a natural *and* inevitable aspect of aging. In this model, individuals were described as gradually progressing from good health to a generalised state of decline, sometimes influenced by underlying genetic aetiology or latent genetic drivers. As one Registrar explained, “I think part of it is nature and happens in everyone. I’m sure there’s a genetic predisposition for becoming more frail more early in life or developing at a later time. I think happens in everyone eventually” (Registrar, P9).

### Theme 2: frailty is a familiar term but it’s meaning is context dependent

Although overall the orthopaedic surgeons in our sample were familiar with the term frailty, they generally described frailty with a degree of uncertainty. This was reflected in the myriad descriptions, reflecting diverse understandings and misunderstandings of the term, but also in the conditions that participants placed on their own statements. For instance, phrases like “*to me”* and *“it’s probably,”* reflected this uncertainty and emphasised relativism in participants’ understanding. Empirical evidence sources were not referred to during the interviews.

The tendency to refer to individual accounts of frailty versus empirical sources reflected that frailty has coexisting dimensions, which result in varied accounts of what frailty “is”. As a Jr. Consultant explained, “I think frailty has a medical and a social sort of concept, doesn’t it, really? I think from a medical point of view we think of frailty meaning a multi-system general degeneration of tissue, which would progress with ageing” (P13). The result of the co-existing social (i.e., lay) and medical (i.e., professional) dimension is that the word “frailty” was used in different ways to communicate different things to different people. Participants acknowledged that the meaning and interpretation of frailty varies by profession and by lay understandings. No participant identified having received education about frailty in specialty training.

For instance, participants used the term frail in a general sense between colleagues to refer to a general state of risk (i.e., term “frail” used as a proxy for risk). 66% of orthopaedic surgeons in our sample stated that they would use the term frailty among colleagues. Registrars (100%) were more likely than Jr. Consultants (50%) or Sr. Consultants (40%) to use frailty in this way. Although the term was used between colleagues, it was regarded as generic rather than a precise clinical term, reflecting “a general public perception rather than a medical terminology” (Sr. Consultant, P3). As one Jr. Consultant expressed: “[Frailty does not enter into the clinical dialogue with colleagues in] terms of the official case notes. It’s not a colloquial word but it is more of a word that we wouldn’t really use. We wouldn’t write it in the notes or anything like that, but certainly colleague to colleague yes, this patient has got a lot of issues they are pretty frail.” (P8, Jr. Consultant).

Participants were less likely to use the term frailty with patients’ families (47%) than with colleagues, and least likely to refer to frailty directly with patients (33%). Jr. Consultants were least likely to use frailty with patients and families (0%). Referring to frailty with patients and particularly families reflected a presumed shared understanding that frailty represented a level of risk that could impact future treatment and outcomes. As a Registrar explained “It gives an idea of their overall risk in terms of what the appropriate treatment from them … [the term] comes up [with patients and families] in terms of talking about rehab and those kinds of things” (P10, Registrar).

How participants understood risk factors for frailty and the relationship between co-morbidities and frailty also differed. Approximately half (47%) of participants discussed an association between fragility and frailty. Osteoporosis, dementia/cognitive decline, fractures, and diabetes were recognised as the next most common associations with frailty (33%). Age was regarded as the most important contributing factor (93%), followed by inactivity (53%) and nutrition (47%). Systematic differences between the subgroups were not observed.

### Theme 3: frailty is generally understood as preventable and reversible, but only under certain conditions

Participants generally regarded frailty as a reversible, or at least a malleable condition if the right strategies were used in the right contexts and at the right time. An important condition to reversibility was a belief that improving frailty requires intervention. This perspective was held by 73% of participants, was most common among Sr. Consultants (100%), and least common among Registrars (50%). Participants (73%) emphasised physical activity or a combination of physical and mental activity (33%) as critical strategies to preventing and reversing frailty; however, none of the Registrars in our sample explicitly mentioned mental activity. Overall, interventions identified were generic, such as exercise and diet or optimization of physical and mental health (Sr. Consultant, P4); however, other factors such as social engagement (Jr. Consultant, P13; Sr. Consultant P3), mobility aid provision (Registrar, P15), medication management (Jr. Consultant, P8), bone health modification (Jr. Consultant, P7) and multidisciplinary team involvement were also identified (Sr. Consultant, P4; Registrar, P12; Jr. Consultant, P13). Improving nutrition (e.g., through meal planning; Registrar, P12) was also identified as an important reversal priority/intervention component (33%), and was most commonly identified by the Registrar subgroup.

A second condition for the modifiability of frailty related to participants mental model; the reversibility of frailty was linked to a perspective of frailty as a “vicious cycle”. Frailty was not modifiable when regarded as an inevitable result of aging. Half of the Registrars (P9, P14, P15) and one Jr. Consultant (P2) regarded frailty as irreversible. However, they believed its progression could be slowed (Jr. Consultant, P2) or “optimised into areas that make them fragile” (Registrar, P14) if recognised and addressed early. Among these participants, early detection was thought to enable frail patients to reduce their risk of future injury and maintain their activity levels for a longer duration (e.g., Registrar P9). Participants who saw frailty as irreversible after a certain point (i.e., Registrars, P9, P14, P15; Jr. Consultant, P2) generally reflected that this understanding was influenced by their clinical experiences of treating vulnerable older patients (e.g., “it comes from clinical experience, every day sort of working, and terms that you hear people use around you”, Registrar, P15). As one Registrar clarified, “the approach that we often have with quite frail people who’ve had hip fractures is that there’s never any goal of getting them better than they were before their fracture. I think that’s … if that was going to be realistic it would be a long, long term thing and I think it’s probably not realistic at all but the goal is to try and get them back to their level of activity that they had before but I think what would actually make more gains is to intervene earlier on” (P9). Participants who described frailty as reversible (66%) generally stated that it is possible to reduce frailty levels in some patients, especially in the early stages, although there was little agreement about how soon intervention should occur. Whether or not frailty was reversible was also tied to circumstances, namely, whether patients have reached “a critical level of accumulated dysregulation and deficits”, which would make reversing frailty “unrealistic” (Registrar, P9). Although participants largely regarded frailty as reversible, many participants were unsure about the distinct role of orthopaedic surgeons in frailty management, prevention, and reversal.

The idea that frailty is generally used and implicitly understood suggested to some participants that education and awareness about frailty is needed. Education could help identify at-risk persons earlier in order to modify a particular frailty trajectory; health education was regarded as necessary for healthcare professionals, patients, and their families. As one Registrar expressed: “I think the main stream is education at different levels, and that also includes patient and patients’ families’ education. And I think involving the primary care physicians within the role is very important” (Registrar, P12). Another Registrar (P11) identified public health education and awareness campaigns for clinicians as useful initiatives, but saw the “lack of glamour” associated with the topic of frailty (in comparison to other public health issues like breast cancer) as a possible barrier to implementation.

### Theme 4: formal screening has utility but its value in orthopaedic practice is unclear

Participants had positive attitudes towards frailty screening in principle (73%), but generally regarded screening as unlikely to be feasible, practical, or useful in orthopaedic practice contexts. A number of factors impacted this perceived utility. Among these were a reliance and trust in non-validated measures such as visual assessment methods and hunches, perceptions of responsibility for screening and alignment with the orthopaedic surgeons’ role, perceived misalignments between frailty screening and the context of orthopaedic practice (e.g., timing of seeing patients, patient status), and concerns regarding the relevance of formal screening to practice settings. Attributes of the screening tools were also pertinent to the perceived relevance and utility of frailty screening in orthopaedic practice contexts. Sr. Consultants most often expressed negative perspectives or indifference towards frailty screening in orthopedic practice (50%). One participant was quick to recognize the influence of frailty on patient management within his speclialized practice, but regarded frailty management as largely outside of his professional scope. Others, such as a Jr. Consultant (P13), expressed that frailty screening in orthopedic practice is only useful if directly linked to organized change for clinical benefit.

Views on whether frailty screening would make a difference to practice were mixed. One third of participants believed that frailty screening would make an impact on their clinical practice, one third believed it would not make a difference, and one third were unsure whether it would or would not. There were no differences noted between subgroups. Factors such as the practice context (e.g., trauma, shoulder specialty), the extent of specialization, and practitioners sense of responsibility (or not) for screening influenced whether frailty screening was regarded as potentially impactful to practice. At times, perceptions of impact related to the fit between the orthopaedic surgeons role and the intent of screening, (e.g., “I don’t feel that orthopaedic surgeons should be the ones who are doing that screening for this patient group” (Jr. Consultant, P7). Other times, perceptions of impact related to the perceived ability of the practitioner to do something with the result, “I would basically allocate the task of assessing for frailty, and doing the appropriate referrals, and involving the appropriate people to my Jr. colleagues, and to the nursing staff, who tend to be able to coordinate this care a bit better than myself” (Registrar, P12).

Although numerous factors were identified that impacted the perceived usefulness of frailty screening in orthopaedics, a reliance on visual screening, often in combination with a patient history, underpinned many participants perspectives. This undermined the apparent value of objective screening. As a Registrar stated, “Potentially, I can’t really see it change at the moment because I kind of come up with my own conclusion that they are frail and that already plays a part.” (Registrar, P14)”. Similarly, a Sr. Consultant expressed that he could “say who’s frail and who isn’t without having to go through a 20-point questionnaire” (P4). While others, such as a Sr. Consultant (P1), recognized that formal frailty screening could assist in predicting treatment outcomes, the vast majority of participants across the subgroups relied on other indicators such as a general vibe, instinct, or impression (P2, P6, P12, P13, P15); “lots of little signals” (P1); age and physical appearance (P11); and history (P2) – often in combination with perception or visual assessment (e.g., P5, P10, P13, P14) – in determining frailty status. These indicators, rather than a quantitative measure or score, reflected an “overall picture” (P8) or “general impression” (P9, 15) by which to identify frailty. Many participants recognized the likelihood that they were under-recognizing frailty by relying on these approaches and some participants, including a registrar and a Sr. consultant, were unaware of the existence of frailty screening tools at all (P6, P10). No differences between the sub groups were noted in participant approaches to identifying frailty.

In addition to needing to link frailty screening with care coordination and effective interventions, participants identified possible benefits of frailty screening in orthopaedic contexts. The most commonly emphasised benefit was an improved ability to predict a patient outcome, expressed by a Sr. Consultant when he stated: “My feeling is that being on the receiving end of issues, that a frailty score would help to … predict the outcome of our treatment” (P1,). The link between frailty status and predicting outcomes from treatment was emphasised (80%) over frailty prevention (27%) and reversing frailty (13%). Preventing adverse outcomes (such as falls and fracture), coordinating care, and guiding intervention planning were also identified as important.

Only two participants (Registrar, P12; Jr. Consultant, P2) explicitly stated that it would be useful or feasible to conduct frailty screening in the orthopaedic practice context. Conversely, general practice was identified as the optimal location for screening by 80% of participants. If frailty screening were to occur in orthopaedic contexts, participants identified simplicity (40%), feasibility and accuracy (27%), as the most important attributes of a screening tool. When the frailty screening tools were ranked, The Frail Questionnaire was most commonly ranked as the preferable screening measure based on its feasibility for use in the orthopaedic practice context (6 preferential rankings). Only the Gait Speed test and PRISMA 7 did not receive a first place ranking. Using the composite scoring method, the GFI (46 points) marginally outscored the Edmonton Frail Scale (47 points), Kihon Checklist (48 points) and Frail Questionnaire (48 points). We triangulated the results of the rank-order and composite-scoring methods to determine the tool most favorably viewed by our participant sample: the Frail Questionnaire (Table 1). The Edmonton Frail Scale, GFI and Kihon Checklist were also viewed relatively favorably by our sample. Participants generally viewed purely physical measures unfavorably for use in orthopaedic practice contexts largely based on attributes on the patient population.

**Table 1:** Feasibility Scoring of Frailty Screening Tools

	Kihon Checklist	Gait Speed	Frail Questionnaire	Edmonton Frail Scale	GFI	PRISMA 7	Timed up and Go
Composite Ranking	48	86	48	47	46	55	87
Inverse Conversion of Composite	1	−1	1	2	3	0	−2
Rank Order Preference	3	0	6	3	2	0	1
Total Score (Inverse + Rank Order)	4	−1	7	5	5	0	−1

## Discussion

Managing frailty, fragility, and multi-morbidity in older patients who at a higher risk of poor outcomes such as falls, fracture, perioperative complications, and readmission is an increasingly critical aspect of orthopaedic practice [19–21, 26–32]. Yet, how frailty is understood and how the use of validated frailty screening tools could aid identification and augment integrated care in this context is markedly limited. In this study, we explored the perceptions of South Australian orthopaedic surgeons towards frailty and frailty screening. We described four dominant themes that summarise participants’ understandings of frailty, the utility and value of frailty screening, and the feasibility of implementing a selection of validated frailty-screening tools.

Orthopaedic surgeons in the sample generally described frailty as a complex and age-related multidimensional state involving both physical and psychological components, associated with increased dependency. Although many participants were able to provide a definition of frailty, the same participants were also less able to identify evidence-based approaches to identification and management. Interventions were discussed generally and seen as “simple” by some, which does not reflect the complexities of older persons adhering to current management recommendations for frailty [33, 34] (e.g., functional, motivational and resource related challenges in conducting strength, cardiovascular and balance exercise activities in conjunction with sufficient protein intake). These findings pointed to a discrepancy between how declaratively participants – particularly Registrars – defined frailty and how this knowledge was displayed and operationalised in the remainder of the interviews. This suggests fundamental differences between the participants’ propositional (information, knowledge-*that*) and operational knowledge (abilities and skills, knowledge-*how*-to [35, 36];) about frailty, and suggests that education emphasizing integrated orthogeriatric management and comprehensive geriatric competencies (e.g., knowledge of frailty and related conditions with reduced physiologic reserve, such as sarcopenia; operational knowledge of psycho-social assessment [31];) may be beneficial to optimizing the care of older persons in orthopaedic contexts. Participants with access to orthogeriatric services were quick to identify the benefit of the assessment and supportive management provided in the care and optimization of older persons with frailty.

The state of knowledge about frailty held by many participants also reflected lay understandings of frailty rather than medical understandings as expressed in academic literature [37]. This was evidenced by how the term was used as a generic reference with patients and colleagues, wherein “frail” was used non-specifically to refer to a general state of risk. A greater knowledge and understanding of the specific components that contribute to frailty or improve intrinsic capacity [38], and a shift towards a perspective of frailty as a continuum rather than a static disease state with loss of capacity for health are important to achieving an evidence based understanding that aligns with global leadership on frailty and its management [39].

Among orthopaedic surgeons in our sample, there was an assumption of a shared understanding of frailty when the term was used colloquially; however, the diverse perspectives and understandings of frailty expressed by participants challenge this taken-for-granted belief. Participants often questioned the clinical relevance of frailty in orthopaedic practice. Frailty was often regarded as an inappropriate term to include in patient notes and was less frequently used in conversations with patients or their families than with other colleagues. Patterns of use and avoidance of the specific term frailty in clinical practice brings into question whether, for some surgeons, there is an associated perception of shame or stigma attached to the term frailty as has been discussed in previous qualitative research in different healthcare contexts [37, 40, 41]. Previous qualitative research suggests that frailty-associated stigma may relate to perceptions of frailty as unmodifiable, determined by individual choice, and inextricably linked to end of life [37]. As such, an evidence-based grounding in what frailty means and its implications for individuals, practitioners and service provision more generally would help generate a shared understanding of frailty between colleagues, may help reduce stigmatization associated with the term frailty, and may facilitate consideration of factors impacting a patients recovery that might otherwise be missed.

Relative to other complex geriatric conditions and considerations such as sarcopenia, falls, and fragility fracture, awareness about frailty is only recently growing and has not formally been included in medical school education or advanced orthopaedic curricula in many countries. Education that attends to the biological, psychological and social components of frailty in diverse orthopaedic practice contexts would likely better equip practitioners to consider the holistic needs of patients during pre and post surgical optimization, and may provide a foundation for integrated and person-centered care of frail older persons [31, 42]. Collaborative, interdisciplinary and multi-level education regarding the merits of frailty screening pertinent to orthopaedic practice and linking with fragility, with an explicit emphasis on the contextual nuances relevant to diverse orthopaedic practice contexts, could therefore be of benefit, if supported by compelling evidence. Although changing current orthopaedic practice would represent a major translational challenge, support from accrediting bodies by way of continuing professional development and education activities and incentives would be a necessary facilitator. General training education that reflects the collaborative necessities and ideals of management (e.g., orthogeriatric collaboration and fracture liason services [31];) with general community understandings may be well placed to attend to challenges of frailty identification and management within and beyond orthopaedic practices. Ideally, such education would be positioned within accepted models of care, particularly those that integrate frailty recognition and defined care responsibilities within orthopaedic practice as part of preoperative assessment, perioperative management, and rehabilitation [43].

Although participants generally saw value in frailty screening for other practice contexts and most often for general practice, they did not use validated measures to recognise frailty. The general assumption was that a frail person could be recognised visually, by way of a general impression, history or clinical assessment – which rendered a formal screening tool unnecessary – and that orthopaedic practice is not a suitable location for screening to occur, given the clinical practice focus. Orthopaedic practice was often seen as too downstream for frailty screening to be of benefit; early intervention and the possibility of screening was endorsed in principle, but was seen as impractical and of little clinical benefit. This is problematic given that frailty, regardless of the measure, is an independent risk factor for adverse health outcomes across surgical specialities, including orthopaedic practices [44, 45]. In contrast, participants in our sample often identified primary care as the most suitable context for the implementation of frailty screening, prevention and management strategies, in alignment with previous research [37]. Further, participants shared the view that frailty ought to be detected and managed by general practitioners (e.g., by deriving an eFI from electronic health records) and community healthcare teams.

Although the majority of participants emphasized the merits of frailty screening in primary care, some participants recognised that a formal frailty screening score could alert to possible issues postoperatively, allow for targeted optimization of risks pre-surgically, and provide a benchmark for future assessment and follow up in orthopaedic practice contexts. This insight is suggestive of a strong potential clinical impact– even rapid frailty assessment tools have demonstrated excellent negative predictive values in surgical contexts [46]. This knowledge could be translated to change orthopaedic surgeon’s perceptions of their professional role in identifying frailty, and could help optimise orthopaedic management plans through integration of assessment and interventions by other disciplines, thereby leveraging the pragmatic application of frailty screening data for clinical benefit.

## Conclusions

As we aspire to more person centred and integrated care for patients with, or at risk of frailty, we see as a prerequisite a shared understanding of frailty, the benefits and limitations of its clinical identification, and the best evidence around its management. The multidimensional nature of frailty with physiological, psychological, socioeconomical and environmental determinants contribute to this challenge, as does a colloquial familiarity with the term and a tendency to rely upon visual assessments for frailty in place of objective measures which are more sensitive to early detection of frailty and pre-frailty. The current study contributes needed perspectives on how a sample of orthopaedic surgeons throughout the life course of practice perceive frailty and frailty screening, highlighting for some the tendency to negate potential impacts of objective screening due to beliefs of the scope of orthopaedic practice; and for others, the potential utility of a frailty assessment to aid in pre-surgical optimization and post-surgical recovery. The findings from this research can help inform future work into complementary and collaborative approaches to recognizing and managing frailty and preventing complications following fracture (e.g., assessing bone health, physical function impairment, and related falls risk). Understanding more about the perspectives of acute care providers like orthopaedic surgeons who frequently care for vulnerable older patients can help address the challenges associated with delivering supportive care for frail patients. This may involve developing strategies to enhance patient safety, guide care planning, and improve transitional care for frail older adults with continuous complex care needs [47].

## Supplementary information

**Additional file 1.** Guide for participants interviews and corresponding ranking data of frailty screening tools.

## Data Availability

The datasets generated and analysed during the current study are not publicly available because participants did not consent to the public release of their data. Further information about the analysis and supportive data is available from the corresponding author on reasonable request.

## Abbreviations

Jr.: Junior
P.: Participant
Sr.: Senior
