# Supplementary material for: Orthopaedic surgeons’ perceptions of frailty and frailty screening
Source: BMC Geriatr. 2020 Jan 16;20:17. doi: 10.1186/s12877-019-1404-8 (PMC6966824; doi:10.1186/s12877-019-1404-8)
Supplement: Supplementary file 1 — Additional file 1. Guide for participants interviews and corresponding ranking data of frailty screening tools. [file 12877_2019_1404_MOESM1_ESM.docx]

**Semi-Structured Interview Questions**

**Part 1: Frailty and Frailty Screening**

- What does the concept of frailty mean to you?
- Where do you think your understanding of frailty has come from?
- Thinking of what frailty means to you, can you offer a definition of frailty from a clinical perspective?
  - When would you say that someone is frail?
  - What is your understanding of how frailty develops? How does it progress?
  - What, if anything, can be done to prevent people from becoming frail? What can be done once a person is frail?
- Do you ever refer to the term frailty in your practice with colleagues? patients? families?
- What can orthopedic surgeons (and other HCPs) do to improve care of pre frail and frail persons?
- Can you describe your attitude towards the concept of frailty screening? Under what circumstances, if any, do you feel it would be useful?
  - Under which time points and settings would it be most useful?
- What would make a screening tool most feasible for use within your practice context?
- If a tool indicated a patient to be frail, what would the logical next steps be for you? Would this change how you already practice, and if so, in what ways?

**Part 2: Views on Selected Frailty Screening Tools**

<*Brief description provided for each tool, followed by general guiding questions>*

Focusing on Tool X now:

1. What are your impressions of this screening tool?
2. What do you think are some of the advantages if any of using Tool X for frailty screening within your practice setting?
3. What do you think are some of the disadvantages if any of using Tool X for frailty screening within your practice setting?
4. Please rank these tools in order of preference
